# Identification of key regulators of pancreatic ductal adenocarcinoma using bioinformatics analysis of microarray data

**DOI:** 10.1097/MD.0000000000014074

**Published:** 2019-01-11

**Authors:** Nan Li, Xin Zhao, Shengyi You

**Affiliations:** a Department of General Surgery, Tianjin Medical University General Hospital; b Tianjin Medical University, Tianjin, China.

**Keywords:** bioinformatics analysis, differentially expressed gene, microarray, pancreatic ductal adenocarcinoma

## Abstract

Pancreatic ductal adenocarcinoma (PDAC) is one of the most lethal forms of cancer, and its etiology remains largely unknown. This study aimed to screen a panel of key genes and to identify their potential impact on the molecular pathways associated with the development of PDAC. Four gene expression profiles, GSE28735, GSE15471, GSE102238, and GSE43795, were downloaded from the Gene Expression Omnibus (GEO) database. The intersection of the differentially expressed genes (DEGs) in each dataset was obtained using Venn analysis. Gene ontology (GO) function and Kyoto Encyclopedia of Genes and Genomes pathway (KEGG) analysis were subsequently carried out. To screen for hub genes, a protein–protein interaction (PPI) network was constructed.

The intersection of the DEGs revealed 7 upregulated and 9 downregulated genes. Upon relaxation of the selection criteria, 58 upregulated and 32 downregulated DEGs were identified. The top 5 biological processes identified by GO analysis involved peptide cross-linking, extracellular matrix (ECM) disassembly, regulation of the fibroblast growth factor receptor signaling pathway, mesoderm morphogenesis, and lipid digestion. The results of KEGG analysis revealed that the DEGs were significantly enriched in pathways involved in protein digestion and absorption, ECM-receptor interaction, pancreatic secretion, and fat digestion and absorption. The top ten hub genes were identified based on the PPI network.

In conclusion, the identified hub genes may contribute to the elucidation of the underlying molecular mechanisms of PDAC and serve as promising candidates that can be utilized for the early diagnosis and prognostic prediction of PDAC. However, further experimental validation is required to confirm these results.

## 1 Introduction

Pancreatic cancer is currently the third leading cause of cancer death; based on the latest cancer statistics, it could lead to approximately 79,400 deaths in China and 330,400 deaths every year worldwide and is predicted to become the second leading cause of cancer death by 2020.^[1,2]^ The median survival of all pancreatic ductal adenocarcinoma (PDAC) patients is less than 6 months, and only 6% of patients are still alive 5 years after diagnosis. Despite the rapid developments in medicine in recent years, the therapies used to treat PDAC are inadequate and its prognosis remains unoptimistic. Moreover, it is almost impossible to detect tumors, as metastasis often take place during the early stages.^[3]^ Accumulated evidence has demonstrated that multiple genes and cellular pathways are closely linked to the occurrence and development of pancreatic carcinoma. Therefore, the development of strategies for early PDAC detection that allow for a surgical cure and increase understanding of the underlying molecular mechanisms that are involved in PDAC is extremely important and desperately needed.

To date, a lack of knowledge regarding the precise molecular mechanisms underlying pancreatic cancer progression has limited the ability to treat advanced disease. There has been widespread research of the genetic biomarkers for PDAC that has utilized high-throughput platforms to analyze gene expression, such as microarrays, that have been increasingly valued as promising tools to identify the genes at the hub of PDAC progression.^[4]^ Previous evidence has identified several genetic markers of pancreatic cancer that have prognostic and therapeutic significance.^[5,6]^ Zhang et al investigated gene-metabolite networks by integrating metabolomics and transcriptomics and demonstrated that impairment of a lipolytic pathway that involves lipases and a unique set of free fatty acids may play an important role in the development and progression of PDAC.^[7]^ Genes having different expression levels in PDAC have been linked to growth factor receptors, tyrosine kinase inhibitors, mitogen-activated protein kinases, the rapamycin blockade, and the phosphatidylinositol 3-kinase and HER2-neu pathways.^[8]^ Additionally, the cell cycle and p53 signaling pathways may play significant roles in pancreatic carcinoma, and differentially expressed genes (DEGs), such as cell division cycle 20, the BUB1 mitotic checkpoint serine/threonine kinase B, cyclin B1, and pituitary tumor-transforming 1, may be potential targets that could be used in the diagnosis and treatment of PDAC.^[9]^ Due to the important role it plays in tumorigenesis, the Ras signaling pathway could also function as an anti-cancer target.^[10]^ Moreover, it has been verified that many other pathways have important effects on tumorigenesis.^[11,12]^ However, the mechanisms underlying the effects of DEGs, particularly those involved in the signal pathways in the association networks implicated in PDAC, remain to be elucidated.

To analyze and enhance understanding of the underlying molecular mechanisms of PDAC, we downloaded microarray data for GSE28735, GSE15471, GSE102238, and GSE43795 from the Gene Expression Omnibus (GEO, http://www.ncbi.nlm.nih.gov/geo/) and identified the DEGs in PDAC tissues and adjacent non-tumor tissues. We identified the common and significant DEGs identified in other studies and selected DEGs present in at least 3 of the microarray datasets for inclusion in gene ontology (GO) and Kyoto Encyclopedia of Genes and Genomes (KEGG) enrichment pathway analysis. Finally, the protein–protein interaction (PPI) networks were constructed. By analyzing their biological functions and associated signaling pathways, this study is likely to shed light on PDAC development at the molecular level and may identify potential candidate biomarkers that may aid in PDAC treatment.

## 2 Materials and methods

### 2.1 Microarray data

The microarray gene expression profile GSE28735,^[5,7]^ GSE15471,^[13]^ GSE102238, and GSE43795^[14]^ related to PDAC were downloaded from GEO (http://www.ncbi.nlm.nih.gov/geo/), which were based on the platform of GPL6244 Affymetrix Human Gene 1.0 ST Array, GPL570 Affymetrix Human Genome U133 Plus 2.0 Array, GPL19072 Agilent-052909 CBC_lncRNAmRNA_V3, and GPL10558 Illumina HumanHT-12 V4.0 expression beadchip, respectively. GSE28735 dataset contained 45 PADC tumors and 45 matching normal pancreatic tissue samples; GSE15471 contained 39 PDAC samples and 39 normal controls; GSE102238 contained 22 PDAC samples and 22 normal controls; and GSE43795 contained 6 PDAC samples and 5 normal controls were selected for subsequent analyses. Ethical approval was not necessary in our study because we downloaded the expression profiles from the public database and do not perform any experiments in patients or animals.

### 2.2 Data preprocessing and DEG identification

The original data were subject to background correction, quantile normalization, and log transformation using the robust multiarray average algorithm.^[15]^ Preprocessing included background correction, quantile normalization, and the transformation of probe IDs into gene symbols. The probes were mapped to genes in NCBI Entrez using the Gene ID converter.^[16]^ If there were multiple probes that corresponded to the same gene, their average value was calculated and used as the final gene expression level.

The raw expression data were screened using the limma package in R/Bioconductor in the GEO2R web tool.^[17,18]^ The *P*-values, which were used to assess the significance of the differences, were calculated using a paired samples *t* test. The obtained *P*-values were adjusted using the Benjamini and Hochberg method.^[19]^ The thresholds used were: an adjusted *P* value < .05, *P* < .05, and |log_2_-fold change|  > 2.0.

### 2.3 Acquisition of the intersection of the DEGs

The intersection of the DEGs, which originated from the 4 expression profiles (GSE28735, GSE15471, GSE102238, and GSE43795), was obtained using Venn analysis.^[20]^ The intersection of the DEGs that originated from the 4 GEO datasets was used in the subsequent analysis.

### 2.4 Functional enrichment analysis of the DEGs

GO analysis (http://www.geneontology.org/) was used to predict the potential functions of the target genes based on molecular function (MF), cellular components (CC), and biological processes (BP).^[21]^ The KEGG (http://www.genome.jp/) is a knowledge base that is used for systematic analysis of gene functions by linking genomic information with higher-level systemic functions.^[22]^ ClueGO is a plugin of Cytoscape that is used to facilitate biological interpretation and to visualize functionally grouped terms in network form.^[23]^ In the present study, we performed KEGG pathway enrichment analysis using the ClueGO plugin with a *P*-value < .05 and a kappa score = 0.4. The DEGs that were found to be enriched within various pathways were visualized using the CluePedia plugin^[24]^ in Cytoscape. For both analyses, a *P*-value of < .05 was considered to denote statistical significance.

### 2.5 Integration of PPI network analysis

The search tool for the retrieval of interacting genes (STRING) database is an online tool that is designed to evaluate PPI information.^[25]^ In this study, DEGs with coexpression coefficients greater than 0.4 were extracted from the STRING database. Thereafter, the PPI networks were constructed using Cytoscape software.^[26]^ The Molecular Complex Detection plug-in (MCODE) was used to screen the PPI network modules in Cytoscape; an MCODE score >5 was used as the selection criterion.

## 3 Results

### 3.1 Identification of DEGs from the 4 selected GEO datasets

We aimed to compare the gene expression profiles of PDAC and normal pancreatic tissues. Four GEO datasets (GSE28735, GSE15471, GSE102238, and GSE43795) were downloaded from the GEO database (https://www.ncbi.nlm.nih.gov/geo/). The main characteristics of these 4 studies are shown in Table 1. We then determined which DEGs were significantly upregulated or downregulated in each study. Compared to the control group, 22 upregulated and 29 downregulated DEGs were identified from GSE28735, 232 upregulated DEGs and 36 downregulated DEGs were identified from GSE15471, 187 upregulated and 89 downregulated DEGs were identified from GSE102238, and 494 upregulated DEGs and 455 downregulated DEGs were identified from GSE43795. The top 10 DEGs from each dataset are listed in Table 2.

**Table 1:**
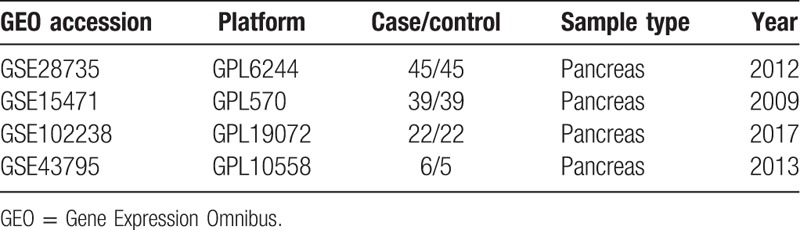
The main features of 4 studies of gene expression microarray data.

**Table 2:**
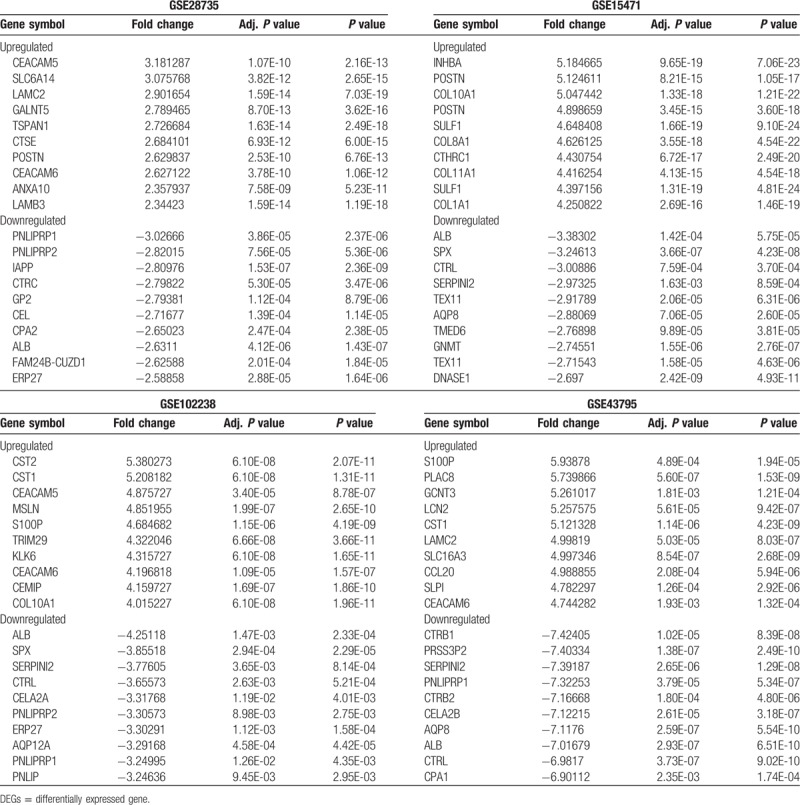
The most significant up- and downregulated DEGs in GSE28735, GSE15471, GSE102238, and GSE43795.

### 3.2 The intersection of the DEGs

**Figure 1:**
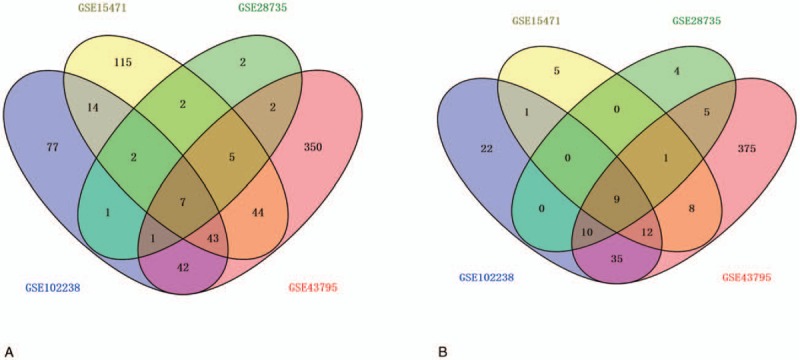
Intersection of the up- and downregulated differentially expressed gene from GSE28735, GSE15471, GSE102238, and GSE43795. (A) Upregulated DEGs. (B) Downregulated DEGs. Each oval represents a study. Numbers in each overlapped area means numbers of differently expression genes in each area. The brown intersection in the middle represents genes which is significantly differentially expressed in 4 microarray datasets consistently. DEGs = differentially expressed gene.

To identify the DEGs that play a role in PDAC, the consistently upregulated or downregulated genes were identified using Venn analysis; a Venn diagram created using Venny2.1.0 is shown in Figure 1. A total of 16 shared DEGs, including 7 upregulated and 9 downregulated DEGs, were found to be consistently and significantly differentially expressed in 4 microarrays.

### 3.3 GO term enrichment and KEGG pathway analysis

GO analysis of the 16 DEGs that were shared across the 4 datasets indicated that they were significantly enriched in the extracellular region and the extracellular space of the CC. The results of the KEGG analysis revealed that the 16 shared genes were enriched in the pancreatic secretion pathway. Upon relaxing the criterion used for selection, we identified more DEGs.

**Figure 2:**
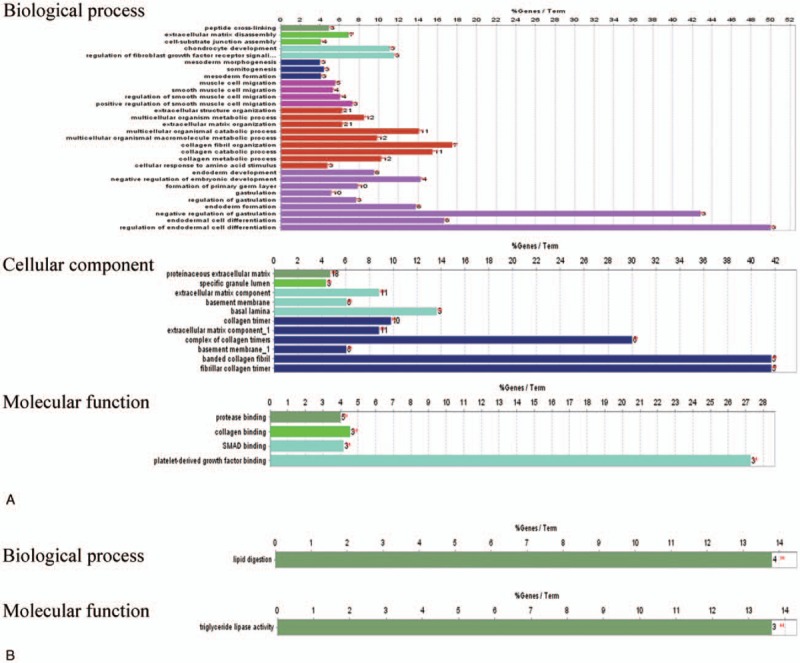
GO enrichment of 4 microarray datasets. GO enrichment analysis of biological process, cellular components and molecular function of upregulated DEGs in at least 3 of microarray datasets. GO enrichment analysis of biological process, cellular components and molecular function of downregulated DEGs in at least 3 of microarray datasets. GO = Gene Ontology.

As shown in Figure 1, 58 overlapping upregulated and 32 overlapping downregulated DEGs in PDAC and control samples were identified in at least 3 of the microarray datasets. Functional enrichment analysis of these DEGs revealed that they were associated with multiple BPs. The GO analysis revealed that the upregulated DEGs were significantly enriched in a number of BP, including peptide cross-linking, extracellular matrix (ECM) disassembly, regulation of the fibroblast growth factor receptor signaling pathway, mesoderm morphogenesis, muscle cell migration, ECM organization, and endodermal cell differentiation; the downregulated DEGs were significantly enriched in BPs involved in lipid digestion. As for CC, the upregulated DEGs were significantly enriched in proteinaceous ECM, the specific granule lumen, ECM components, and collagen trimers. Moreover, GO MF analysis indicated that the upregulated DEGs were significantly enriched in BPs involved in protease binding, collagen binding, and platelet-derivation; the downregulated DEGs were significantly enriched in BPs involved in the triglyceride lipase activity (Fig. 2). The results of the KEGG analysis revealed that the DEGs were significantly enriched in processes involved in protein digestion and absorption, ECM-receptor interactions, pancreatic secretion, and fat digestion and absorption (Table 3).

**Table 3:**
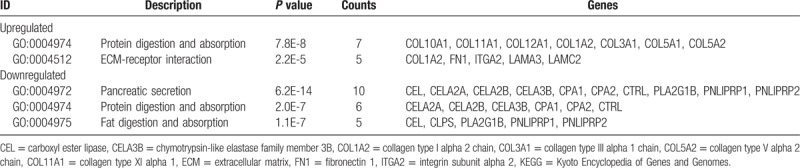
KEGG pathway analysis of differentially expressed genes.

### 3.4 Module screening of the PPI network and pathway enrichment analysis

**Figure 3:**
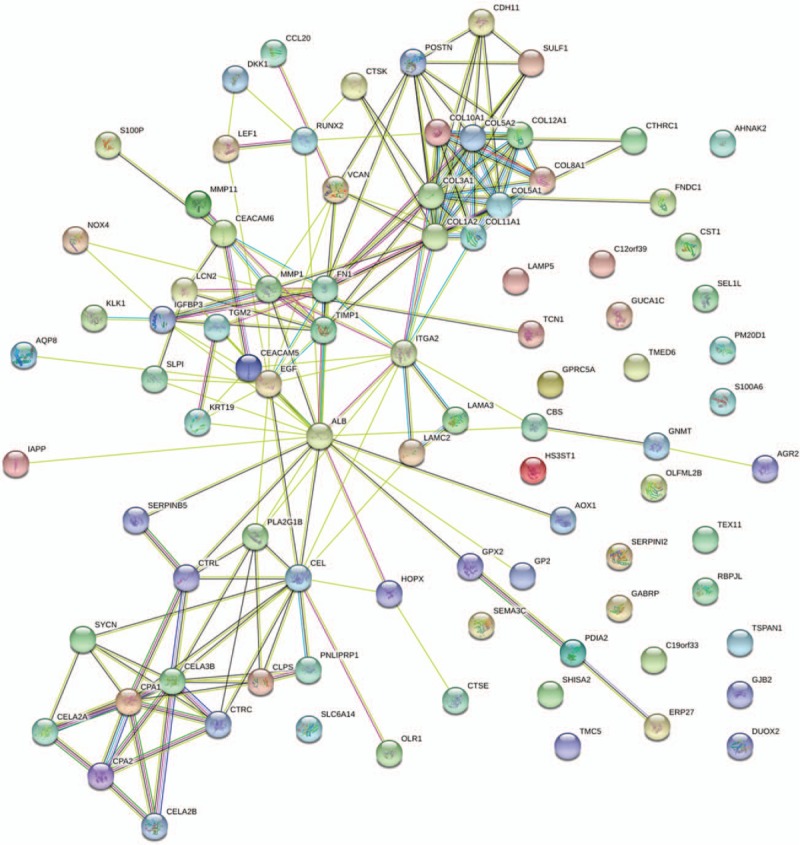
Protein–protein interaction network of differentially expressed genes.

**Figure 4:**
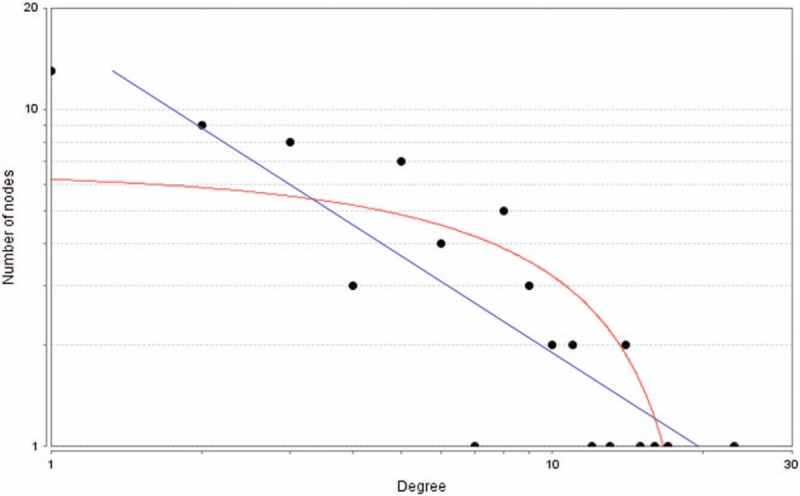
The distribution of core genes in the interaction network. The black node means the core gene. The red line means the fitted line, and the blue line means the power law. The correlation between the data points and corresponding points on the line is approximately 0.908. The *R*-squared value is 0.768, giving a relatively high confidence that the underlying model is indeed linear.

**Figure 5:**
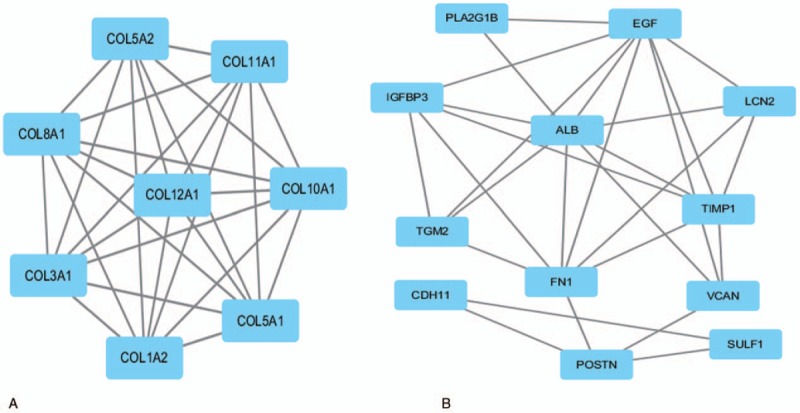
The modules from protein-protein interaction network. The squares represent the DEGs in modules, and the lines show the interaction between the DEGs. DEGs = differentially expressed gene.

Based on information found in the STRING database, the gene interaction network was shown to contain 89 nodes and 183 edges. The nodes represented the DEGs, and the edges represented the interactions among the DEGs. The Network Analyzer in Cytoscape was used to analyze these genes, (Fig. 3) and the core genes were ranked according to the predicted scores. The top 10 high-degree hub nodes included albumin (ALB), collagen type I alpha 2 chain (COL1A2), epidermal growth factor (EGF), collagen type III alpha 1 chain (COL3A1), fibronectin 1 (FN1), carboxyl ester lipase (CEL), integrin subunit alpha 2 (ITGA2), collagen type V alpha 2 chain (COL5A2), matrix metallopeptidase 1 (MMP1), and chymotrypsin-like elastase family member 3B (CELA3B). The core genes and their corresponding degrees are shown in Table 4. The distribution of the core genes in the interaction network is shown in Figure 4; the black node represents the core gene, the red line represents the fitted line, and the blue line represents the power law. The correlation between the data points and the corresponding points on the line was approximately 0.908. The *R*-squared value was 0.768, which indicated with relatively high confidence that the underlying model was linear. Subsequently, MCODE was applied in Cytoscape to screen the modules of the gene interaction network; the top 2 modules are shown in Figure 5.

**Table 4:**
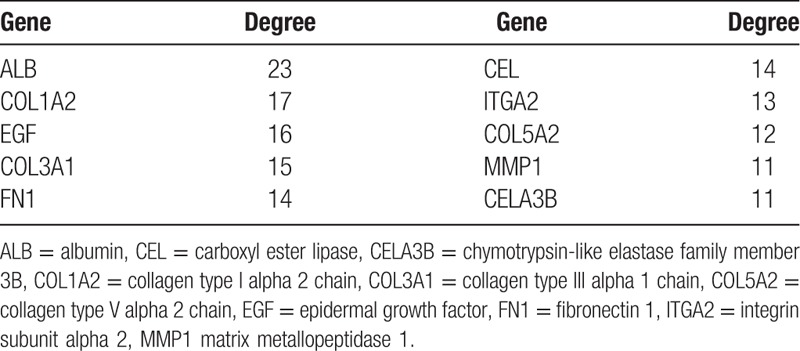
The core genes and their corresponding degree.

## 4 Discussion

Currently, the early detection of pancreatic cancer remains a challenge for clinical scientists, and understanding of the molecular mechanisms underlying the progression of PDAC is still relatively poor. In this study, we utilized functional genomic data to discover targeted pathways and function-based genes, and a bioinformatics method was used to identify the key regulators involved in the development of PDAC.

In the present study, the gene expression profiles GSE28735, GSE15471, GSE102238, and GSE43795 were downloaded and a comprehensive bioinformatics analysis was performed. Using the previously defined cut-off criterion, we identified 16 shared DEGs (7 upregulated and 9 downregulated) in 4 microarrays. We then relaxed the selection criteria, which allowed us to identify more DEGs. GO function and KEGG pathway annotation of the 90 overlapped DEGs identified in at least 3 of the microarray datasets showed that these genes may play vital roles in the occurrence of PDAC. By constructing a PPI network, we also identified several hub genes that are highly correlated with the development of this disease.

GO analysis of the 16 DEGs that were shared across all 4 datasets indicated that these genes were significantly enriched in the extracellular region and the extracellular space of the CC. The results of the KEGG analysis revealed that the 16 shared genes were enriched in the pancreatic secretion pathway. Upon relaxing the selection criteria, we identified more DEGs. Among the 90 DEGs that were identified, the GO term analysis showed that the significant BP-related ontological categories represented by the upregulated DEGs included peptide cross-linking, ECM disassembly, regulation of the fibroblast growth factor receptor signaling pathway, mesoderm morphogenesis, muscle cell migration, ECM organization, and endodermal cell differentiation. Peptide cross-linking is an important BP of proteins with specific functioning; studies have shown that peptide cross-linking is involved in multiple processes that have been implicated in pancreatic cancer development.^[27,28]^ Tumor differentiation, invasion and metastasis are regulated by multiple factors during the multi-step processes that are involved in ECM assembly and degradation.^[29]^ At the molecular level, a high percentage of PDACs overexpress a number of growth factors and their receptors, including members of the EGF, transforming growth factor, and fibroblast growth factor families.^[30]^ Almost all of the downregulated genes have lipid digestion and triglyceride lipase activity; lipid protection and digestion play important roles in cell proliferation and tumorigenesis.^[31]^ A recent review indicated that glycerolipid metabolism plays a prominent role in human physiology and disease via its involvement in fat storage and metabolic disorders as well as cancer survival pathways.^[32]^ It has been reported that protein digestion and absorption are implicated in the development of pancreatic neuroendocrine tumors.^[33]^ An improved understanding of metabolic dysregulation in pancreatic cancer could lead to the discovery of novel therapeutic targets.^[34]^ Therefore, GO analysis could help to identify the BPs involved in the occurrence of PDAC, as well as the related MFs and CCs. Moreover, it may provide evidence of the impact of the functioning of various genes on metabolism and metabolite networks.

The enriched KEGG pathway comprised of 90 DEGs included DEGs involved in ECM-receptor interaction, pancreatic secretion, and protein and fat digestion and absorption. Cancer cells actively take part in the production of ECM proteins, which are then deposited into the tumor stroma. In recent years, studies focused on the tumor microenvironment have suggested that the remodeling of the tumor ECM increases the invasive and metastatic capabilities of tumor cells.^[35]^ Maturity-onset diabetes of the young (MODY) is a genetically defined subgroup of diabetes characterized by an autosomal dominant inheritance and early onset, non-insulin dependent diabetes that is closely related to pancreatic secretory dysfunction.^[36]^ Patients with CEL mutations develop pancreatic exocrine dysfunction in early childhood and eventually develop MODY.^[37]^ Interestingly, a study has demonstrated that CEL-MODY patients may be at an increased risk of developing pancreatic cancer.^[38]^ Collagen type XI alpha 1 (COL11A1) belongs to the collagen family and is a major component of the interstitial extracellular matrix.^[39]^ According to 1 report, high COL11A1 mRNA levels are significantly associated with poor chemo-responses and clinical outcomes in epithelial ovarian carcinoma.^[40]^ In a proton magnetic resonance study, it was found that lipids, choline-containing compounds, and fatty acids were decreased in pancreatic cancer tissues compared with normal pancreatic tissue.^[41]^ Lipids may be protumorigenic, as a high-fat diet was found to promote tumor growth in a murine model of pancreatic cancer.^[42]^ Indeed, lipid metabolism is likely to be an important source of energy in pancreatic cancer. However, it remains to be determined which fatty acids are cytotoxic for tumor cells and which fatty acids provide tumors with metabolic substrates. Further studies of tumor metabolism could reveal exciting possibilities for therapeutic intervention. Thus, a comprehensive understanding of the involved pathways and their internal links may aid us in elucidating the major mechanisms underlying the development of PDAC.

To obtain more evidence, we analyzed the PPI network and found that ALB, COL1A2, EGF, COL3A1, FN1, CEL, ITGA2, COL5A2, MMP1, and CELA3B were the top 10 hub genes, which may be essential to the molecular mechanisms underlying the development of PDAC and may therefore serve as potential therapeutic targets. ALB was identified as one of the hub genes with the greatest degree of connectivity. Pant et al^[43]^ reported that the baseline serum ALB level is a predictive biomarker for patients with advanced PC that are treated with bevacizumab, which may be explained by a pharmacokinetic description of bevacizumab. Researchers found that fibroblast growth factors (FGF) treatment of gynecological cancer cells could promote calpain 2 activity.^[44]^ This finding is consistent with those of a previous study that reported that EGF increased calpain-2 activity via the activation of extracellular signal-regulated kinase pathways.^[45]^ The 2 most extensively studied FGFs are FGF-1 and FGF-2.^[46]^ FGF-2 is the prototypic heparin-binding protein and has growth-related, antiapoptotic and angiogenic activity.^[47]^ It has been found to be overexpressed in PDAC,^[48]^ and the FGF-2 receptor has been reported to be involved in tumor metastasis, stage, and retroperitoneal invasion.^[49]^ Fibronectin (FN), which exists in both plasma and cells, functions in cellular adhesion, spreading, and cell migration.^[50]^ Studies have demonstrated that FN stimulates invasion and adhesion and markedly inhibits cell death in pancreatic cancer cells.^[51,52]^ CEL has been demonstrated to be expressed primarily in pancreatic acinar cells and lactating mammary tissue and encodes a digestive enzyme that has been shown to play a role in cholesterol ester digestion.^[53]^ In addition, Ferraro et al^[54]^ proved that a decrease in ITGA2 could reduce cell migration in colon cancer. Reportedly, CEL-mutation carriers develop multiple pancreatic cysts during the development of diabetes,^[38]^ and these cysts have been shown to promote the occurrence of pancreatic cancer.^[55]^ Genetic analysis has shown that human chymotrypsin-like elastase family member 3A (CELA3A) and CELA3B are involved in complex formation between proelastases and procarboxypeptidases in chronic pancreatitis.^[56]^ It has been reported CELA3A and CELA3B are downregulated in prostate cancer,^[57]^ which is consistent with our results. Fibrosis is a consequence of injury that is characterized by the accumulation of excess collagen and other ECM components that results in the destruction of normal tissue architecture and loss of functioning; COL1A2, COL3A1, and COL5A2 are genes that are involved in the maintenance of the ECM that have been shown to have a close relationship with human dermal fibroblasts.^[58]^ Matrix metalloproteinases (MMPs) are a group of zinc-dependent proteases involved in ECM and basement membrane degradation.^[59]^ A recent study showed that MMP1 activated AKT and induced protease-activated receptor-1-expressing dorsal root ganglia (DRG) to release substance P, which, in turn, activated neurokinin 1 receptor-expressing PDAC cells and enhanced cellular migration and invasion.^[60]^ The results of the studies of the hub genes are consistent with the results of the enriched function and pathway analysis and could provide for effective diagnostic and therapeutic strategies for PDAC. Furthermore, we analyzed the gene interaction network and the top 2 modules using MCODE, and found that COL1A2, COL3A1, and COL5A2 were the core interaction genes for PDAC. This finding was in accordance with our STRING database results.

Collagen is the major component of the interstitial extracellular matrix. The ECM is known to play an active role in numerous BP including those involved in the maintenance of cellular shape, proliferation, migration, differentiation, and apoptosis, as well as carcinogenesis.^[61]^ A previous study found that nano-targeted relaxin impairs fibrosis and tumor growth in pancreatic cancer, reduces collagen I, desmin (hPSC marker) and CD31 (endothelial marker) expression, and improves the efficacy of gemcitabine.^[62]^ High levels of COL1A2 expression are associated with the pathogenesis of systemic sclerosis.^[63]^ Cancer-associated fibroblasts, which are the key effector cells in PDAC, are known to induce tumor growth and progression. It has been reported that COL3A1 is a gene related to adhesion that plays a role in EMT in hepatocellular carcinoma.^[64]^ A previous study has found that molecules involved in ECM remodeling (eg, COL5A2 and MMP13) were differentially expressed in ductal carcinoma in situ and invasive ductal carcinoma in breast cancer.^[65]^ Therefore, we propose that COL1A2, COL3A1, and COL5A2 may play a role in promoting pancreatic fibrosis and epithelial-mesenchymal transition via the ECM-receptor interaction pathway in the early stages of PDAC and may thus serve as indicators of a critical point in disease development. Upon reviewing the relevant literature, no report was found that indicates that these genes play a definitive role in the development of PDAC. Further studies that aim to measure the levels of these genes and confirm their explicit roles in PDAC should be conducted. Improved understanding of the roles of these genes in PDAC would help us to elucidate the crucial mechanisms that underlie tumorigenesis. These genes could potentially serve as targets for the early diagnosis or treatment of this disease.

Although bioinformatics technologies have the ability to identify the potential gene candidates involved in tumorigenesis, several limitations of this study should be noted. First, the sample sizes used for the expression profiling were small and some clinical variables were not available, and thus, we failed to consider factors such as the presence of different cancer states to distinguish driver/casual genes and passenger genes and to determine the availability of diagnostic biomarkers in blood. Second, the results obtained solely by means of bioinformatic analysis are not sufficient, and experimental verification is needed to confirm these results. Therefore, further genetic and experimental studies with larger sample sizes and experimental verification are required.

In conclusion, we identified several core genes and pathways that were closely related to the initiation and progression of PDAC using a series of bioinformatics analyses of DEGs in tumor and normal samples. Identification of the involved genes, COL1A2, COL3A1, and COL5A2, provided greater insight into the specific molecular mechanisms underlying PDAC occurrence and development, especially in terms of the pathways involved in fat metabolism and ECM-receptor interaction. Upon further experimental validation of these results, these genes may serve as potential research targets for therapy and thereby contribute to greater understanding of the molecular mechanisms underlying the development of PDAC.

## Author contributions

**Formal analysis:** Nan Li.

**Investigation:** Nan Li.

**Methodology:** Nan Li, Xin Zhao.

**Software:** Xin Zhao.

**Supervision:** Shengyi You.

**Writing – original draft:** Nan Li.
